# Application of Artificial Intelligence Models to Predict the Onset or Recurrence of Neovascular Age-Related Macular Degeneration

**DOI:** 10.3390/ph17111440

**Published:** 2024-10-28

**Authors:** Francesco Saverio Sorrentino, Marco Zeppieri, Carola Culiersi, Antonio Florido, Katia De Nadai, Ginevra Giovanna Adamo, Marco Pellegrini, Francesco Nasini, Chiara Vivarelli, Marco Mura, Francesco Parmeggiani

**Affiliations:** 1 Unit of Ophthalmology, Department of Surgical Sciences, Ospedale Maggiore, 40100 Bologna, Italy; dr.fsorrentino@gmail.com (F.S.S.); c.carola@live.it (C.C.); antonioflorido@outlook.it (A.F.); 2 Department of Ophthalmology, University Hospital of Udine, 33100 Udine, Italy; markzeppieri@hotmail.com; 3 Department of Translational Medicine and for Romagna, University of Ferrara, 44121 Ferrara, Italy; katia.denadai@unife.it (K.D.N.); ginevragiovanna.adamo@unife.it (G.G.A.); marco.pellegrini@unife.it (M.P.); chiara.vivarelli@unife.it (C.V.); marco.mura@unife.it (M.M.); 4 ERN-EYE Network-Center for Retinitis Pigmentosa of Veneto Region, Camposampiero Hospital, 35012 Padua, Italy; 5 Unit of Ophthalmology, Azienda Ospedaliero Universitaria di Ferrara, 44100 Ferrara, Italy; francesco.nasini@ospfe.it; 6 King Khaled Eye Specialist Hospital, Riyadh 12211, Saudi Arabia

**Keywords:** neovascular age-related macular degeneration, artificial intelligence, deep learning, retinal biomarkers, therapy prediction

## Abstract

Neovascular age-related macular degeneration (nAMD) is one of the major causes of vision impairment that affect millions of people worldwide. Early detection of nAMD is crucial because, if untreated, it can lead to blindness. Software and algorithms that utilize artificial intelligence (AI) have become valuable tools for early detection, assisting doctors in diagnosing and facilitating differential diagnosis. AI is particularly important for remote or isolated communities, as it allows patients to endure tests and receive rapid initial diagnoses without the necessity of extensive travel and long wait times for medical consultations. Similarly, AI is notable also in big hubs because cutting-edge technologies and networking help and speed processes such as detection, diagnosis, and follow-up times. The automatic detection of retinal changes might be optimized by AI, allowing one to choose the most effective treatment for nAMD. The complex retinal tissue is well-suited for scanning and easily accessible by modern AI-assisted multi-imaging techniques. AI enables us to enhance patient management by effectively evaluating extensive data, facilitating timely diagnosis and long-term prognosis. Novel applications of AI to nAMD have focused on image analysis, specifically for the automated segmentation, extraction, and quantification of imaging-based features included within optical coherence tomography (OCT) pictures. To date, we cannot state that AI could accurately forecast the therapy that would be necessary for a single patient to achieve the best visual outcome. A small number of large datasets with high-quality OCT, lack of data about alternative treatment strategies, and absence of OCT standards are the challenges for the development of AI models for nAMD.

## 1. Introduction

Currently, we categorized age-related macular degeneration (AMD) into two subsets: wet AMD, also known as neovascular AMD (nAMD), and dry AMD [1]. nAMD is typically characterized by the onset of new blood vessels, namely neovascularization, in the subretinal or intraretinal layers. If unchecked, neovessels can cause bleeding, edema, and eventually blindness [2]. The conventional therapy for nAMD consists of administering intravitreal injections of anti-vascular endothelial growth factor (VEGF) medicines for a long time to inhibit and reduce the formation of these newly formed blood vessels [3]. Different treatment regimens, such as regular intervals of 4-7 treatments, as-needed basis Pro-re-nata (PRN) injections, and treat-and-extend (T&E) injections, are used based on the retreatment approach. Usually, the further treatment plan is determined after three first injections, with an increasing tendency to employ the T&E approach in order to minimize vision impairment caused by relapse [4]. Nevertheless, the use of T&E may include the potential danger of excessive treatment depending on the patient’s condition.

AI is a comprehensive term that includes many computing methods, such as machine learning (ML), which pertains to algorithms that derive insights from data, and deep learning (DL), a subset of ML that employs deep neural networks for intricate pattern recognition in data [5].

## 2. Treatments for Neovascular Age-Related Macular Degeneration

**Figure 1:**
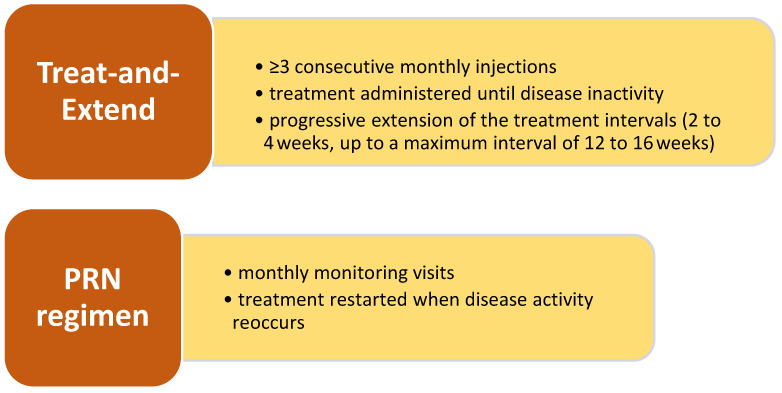
Methods to treat neovascular age-related macular degeneration.

Administered on a fixed treatment schedule, several anti-VEGF intravitreal therapies (IVT), such as bevacizumab, ranibizumab, brolucizumab, aflibercept have become widely accepted treatments for nAMD [6]. To achieve an ideal outcome, it is necessary to provide multiple injections into the eye at scheduled time intervals. In clinical studies, the average improvement in vision from the starting point was around 7 to 11 letters after one year, accomplished with a total of approximately 7.5 to 12 injections of IVT. However, the average patients in real-world clinical practice are not reaching these visual outcomes [7]. Several causes have been linked to this, with notable emphasis on disparities between patient demographics in real-world settings and those in clinical trials, as well as variations in treatment frequency. The point is that the intervals and frequency at which a dose is administered are consistently linked to vision outcomes (Figure 1). Real-world studies have reported an average vision improvement from 0 to 3 letters after one year, accomplished with approximately 5 to 7 injections of IVT [8].

Multiple investigations have found that baseline visual acuity is a reliable indicator of long-term visual outcomes [9]. It is commonly believed to have a sort of correlation with the severity of the disease and the anatomical alterations in the neurosensory retina. Several anatomical characteristics have been linked to poorer vision outcomes, including choroidal neovascularization (CNV), interruption of the external limiting membrane, disruption of the ellipsoid zone, intraretinal fluid (IRF), subretinal fluid (SRF), and increased choroidal thickness [10]. During the first year of fixed monthly/bimonthly dosing, patients with occult CNV, presence of retinal fluid, and fluorescein leakage were less likely to reach every 12-week dosing in the second year of treatment frequency [11].

In the clinic, physicians make decisions regarding the course of care for each patient despite the fact that treatment paradigms are typically established based on the average treatment response of a cohort. The primary concern is the identification of the most effective treatment plan for each individual, with a focus on the least amount of inconvenience and the highest prospective visual improvement [7]. This continues to be unpredictable and difficult because of the necessity of multiple IVTs and the variable response to various anti-VEGF molecules in real-world scenarios. In the HARBOR clinical trial, patients who were adhering to a PRN regimen were administered a treatment regimen that included a range of injections, from 3 to 24, over a two-year period. The distribution of the quantity of injections was nearly uniform. During the second year of the VIEW clinical study, more than 50% of patients had PRN treatment intervals of at least 12 weeks. The eyesight outcomes of these patients were comparable to those of those who required more frequent therapy [12]. Although conventional analyses that are predicated on conventional imaging evaluations offer valuable insights at the population level, they have not had a substantial influence on the treatment decisions of individual patients.

The field of diagnosing and treating retinal maladies has made substantial progress as a result of the application of AI, which has led to a plethora of research discoveries. A predictive model has been developed to predict whether the injection interval for administering T&E using anti-VEGF medications for nAMD treatment will be less than 5 weeks (indicating a high treatment burden) or more than 10 weeks (indicating a low treatment burden) [13]. Anticipating the individuals who will require urgent T&E treatment due to the recurrence of nAMD within three months of receiving their three initial injections, identifying those who can tolerate a longer interval between treatments, and identifying those who may opt for PRN treatment after three months will aid in the planning of treatment [14]. The future is the development of an AI model that can predict which group of nAMD-naïve patients will experience a recurrence within three months of confirming dryness following the first injections.

## 3. Artificial Intelligence Strategies

Machine learning (ML) is a subfield of AI that comprises training a machine to identify specific patterns within an extensive dataset because of the utilization of a wide range of interconnected algorithms layered together, with each algorithm dedicated to identifying specific traits [15]. This system is known as a neural network because it tries to replicate the functioning of neurons in the human brain. Deep learning (DL) is a branch of ML that involves the use of many artificial neural networks (ANNs) arranged in layers to more accurately imitate the processing skills of the human brain. Convolutional neural networks (CNNs) are specific types of ANNs that are extensively employed with the aim of analyzing videos and images. The programs’ successful data interpretation can be quantified using measures such as specificity, sensitivity, or a receiver operating characteristic (ROC) curve [16]. The ROC curve represents the relationship between the true positive rate and the false positive rate. Over the last twenty years, there has been a significant increase in the use of AI-driven solutions in the medical field [17].

AI and ML algorithms are commonly trained using digital photos and numerical data. Largely used in healthcare for quick analysis of imaging, DL is highly effective in handling high-dimensional data without the need for manual feature engineering. DL has demonstrated promise in identifying referable age-related macular degeneration (AMD), with research studies indicating satisfactory diagnosis accuracy. Although many DL systems have undergone training and testing using extensive datasets such as AREDS, additional research is needed to validate and generalize their performance across diverse populations and imaging modalities. Within the field of OCT imaging, deep learning has facilitated the automatic categorization of AMD and the precise division of retinal structures with exceptional precision [18]. The utilization of DL frameworks such as U-Net has enhanced the process of boundary and feature-level segmentation, making it easier to detect disorders such as choroidal neovascularization and macular edema.

AI has the potential to offer numerous benefits in the management of nAMD. In terms of accuracy, AI methods (CNNs, DL, and ML), particularly CNNs and DL algorithms, have demonstrated a high degree of precision in the identification of early macular changes from imaging techniques such as fundus photographs and OCT scans. AI models have achieved AUCs, sensitivities, and specificities that exceed 90%, as evidenced by a multitude of research studies. Despite the fact that retinal imaging is well-established, its precision may be affected by the patient’s compliance and the examiner’s expertise. AMD is often detected at a later stage of the disease, after significant damage has already occurred, using conventional methods. In terms of efficiency, AI models demonstrate exceptional efficiency in the processing of extensive imaging data, which enables the expedition of diagnoses without the need for tedious manual evaluations. AI has the potential to reduce the workload of physicians significantly and enable the earlier detection of nAMD by automating standard screening procedures. In a matter of seconds, AI-driven systems can assess fundus photographs or OCT scans, all while simultaneously providing reproducible and dependable results. This facilitates improved scalability, particularly in the context of extensive screening initiatives and telemedicine. Conventional methods and traditional nAMD detection techniques, such as OCT and angio-OCT assessments and manual imaging analysis, are more time-consuming. Additionally, the duration of diagnosis and care is often extended by the necessity of retesting for validation in traditional procedures.

## 4. AI-Driven Identification of Retinal Biomarkers

This section examines the function of artificial intelligence in detecting essential retinal biomarkers, including IRF, subretinal fluid (SRF), and pigment epithelial detachment (PED), which are vital for diagnosing and tracking the progression of age-related macular degeneration.

It is anticipated that the global prevalence of nAMD will experience a significant increase by 2050, affecting approximately 300 million individuals [19]. The demand for ophthalmology services is currently exceeding the financial and personnel resources required to maintain high-quality and long-lasting services, resulting in unprecedented capacity issues. The development of more effective and secure methods for diagnosing and treating AMD is imperative because of an increase in patient demand and the constraints of physicians [20]. This is essential to guarantee that patients receive the necessary treatment, prevent the superfluous progression of the illness, and, in the end, prevent vision loss.

Artificial neural networks (ANNs) are algorithms that assist in the identification of patterns and intricate structures in vast datasets derived from retinal imaging. The ability of convolutional neural networks (CNNs) to adapt and improve based on prior experiences is a critical characteristic. This allows them to become sophisticated instruments for forecasting and categorization by accepting a variety of inputs. By incorporating AI technology into ophthalmology services, the anticipated burden on public hospitals and health services can be alleviated [21].

In the field of ophthalmology, research has demonstrated that AI is capable of identifying retinal disease from OCT retinal images with a level of sensitivity and specificity that is comparable to that of a physician. The reported accuracy rates range from 90% to 95%. AI models are capable of accurately categorizing retinal images that exhibit a variety of conditions, including AMD, drusen, mottling RPE, choroidal neovascularization, and healthy retinas, with an accuracy rate of over 90%. Nevertheless, the diagnosis and monitoring of AMD are not adequately addressed by relying solely on OCT retinal images. Clinicians would be significantly aided in their monitoring approach by the precise identification of retinal biomarkers that indicate disease activity in AMD [22]. The optimization of treatment regimens and the prediction of therapy response remain significant challenges for patients with nAMD. Instruments that are currently available have the potential to enhance confidence in the clinical development of novel therapies, facilitate the creation of individual prognostic forecasts, and ultimately provide information that is highly beneficial for treatment decisions during clinics, all of which are powered by AI.

## 5. Artificial Intelligence Models

This section examines DL algorithms, a category of ML, and their utilization in OCT image analysis for forecasting therapeutic response in nAMD.

The following are the examples of AI, DL, and ML models discussed hereby:

- CNNs: extensively employed in numerous studies for the analysis of imaging data, including fundus pictures and OCT scans, exhibiting significant sensitivity and specificity in identifying macular alterations;
- Random Forests and Support Vector Machines (SVMs): these models are frequently employed for classification tasks, distinguishing between healthy and eyes affected by AMD based on certain clinical criteria, including the thickness of the retinal neuroepithelium or the presence of intra- or subretinal fluid;
- Bayesian Networks: utilized in various instances to amalgamate several diagnostic tests and clinical data, yielding probabilistic results to evaluate macular exudation risk;
- Deep Learning Algorithms: models utilizing fundus imaging and OCT have demonstrated efficacy in the early detection and ongoing monitoring of AMD;
- Explainable Artificial Intelligence (XAI): this is becoming an essential method for improving the interpretability of AI models, hence rendering clinical decision-making more transparent and reliable for practitioners.

Deep learning is a subset of artificial intelligence where many resources are now invested to try to identify predictive indicators for individualized outcomes. Nevertheless, the majority of progress in the recent intensive use of AI for nAMD has focused on creating models that support eye specialists in image analysis [4]. These algorithms are designed to implement and automate the process of segmenting, extracting, and quantifying imaging-based features from the imaging acquisition starting from the optical coherence tomography (OCT). Typically, the process of training, fine-tuning, and testing AI algorithms necessitates the use of extensive and high-quality datasets [23]. With comparatively few datasets, several research groups have created AI-based algorithms for nAMD. Notably, among these datasets, the Moorfields Eye Hospital real-world AMD database and HARBOR stand out in the literature [24]. The phase 3 HARBOR study (NCT00891735) evaluated the effectiveness of ranibizumab in 1097 patients with treatment-naïve nAMD. The trial compared two different dosages of the drug and two treatment regimens: monthly and PRN treatment. Significantly, HARBOR was the initial prominent clinical trial for nAMD to employ spectral-domain OCT, enabling precise extraction of high-sensitivity features [25]. Moorfields Eye Hospital, a specialized retinal center in the United Kingdom, has a comprehensive database of electronic medical records and OCT images. This database includes information from patients with AMD who have received at least one injection of ranibizumab or aflibercept between 2008 and 2018 and have been followed up for at least 1 year [26]. The Moorfields AMD dataset has a total of 8174 eyes belonging to 6664 patients. A de-identified version of the segmentation results is publically accessible to the research community [27,28].

Using a DL-based framework with two independent networks, De Fauw and colleagues developed a critical model for OCT image segmentation and disease classification [28]. This model is used to conduct automated diagnosis of retinal diseases on OCT scans. This approach has been used to study imaging biomarkers and visual outcomes [29]. Subsequently, a different group created an innovative automatic segmentation model utilizing CNNs [30]. The construction of this model involved the utilization of a substantial and authentic dataset derived from electronic medical records in the United Kingdom. This dataset was meticulously annotated by clinical specialists, who identified and labeled the most prevalent biomarkers associated with AMD on OCT scans. The retinal biomarkers comprise pigment epithelial detachment (PED), intraretinal fluid (IRF), and subretinal fluid (SRF) [30]. The Notal OCT Analyzer and the Medical University of Vienna AI-based Fluid Monitor are advanced instruments that can automatically detect and measure fluids in OCT images [31,32]. In particular, these have helped to more accurately measure and map changes in IRF and SRF over time, as well as to conduct quantitative measurements across several big datasets and to investigate questions about retinal fluid measures and visual outcomes [33,34].

For example, when the Notal OCT Analyzer was used on a real-world dataset, it showed that higher changes in some measurements (IRF, SRF, PED, central subfield thickness, and total fluid) during the anti-VEGF maintenance phase were linked to poorer visual acuity after 2 years [34,35]. Some preliminary investigations utilizing the AI-powered Fluid Monitor demonstrated distinct effects of SRF and IRF on visual outcomes. In both the FLUID and HARBOR trials, higher volumes of IRF, but not SRF, were found to have a negative correlation with visual acuity [24]. Specifically, for every 100 nanoliter increase in IRF volume, visual acuity decreased by an average of -4.00 and -2.84 letters, respectively. On the other hand, an increase in SRF volume by 1.10 and 1.43 (which was not statistically significant) did not have a significant impact on visual acuity. The range of numbers is from 32 to 33, inclusive. Using the approach used by De Fauw, the Moorfields AMD database revealed a more significant correlation between IRF and visual acuity compared with SRF [28]. Additional research has utilized AI models to analyze OCT images for various research purposes. These include predicting visual acuity from OCT, extracting advanced features such as ellipsoid zone integrity and subretinal hyperreflective material volume, enabling correlation analysis among multiple OCT features, comparing different types of eye conditions, and grouping patients based on characteristics related to CNV using unsupervised ML.

## 6. Artificial Intelligence and the Therapy Prediction

This part explores AI models developed to forecast individualized therapy needs and response rates, utilizing OCT biomarkers previously revealed in the article, building upon the uses of AI in diagnosis and prognosis.

Algorithms for forecasting therapy response and required treatment frequency have been started to be developed by several research groups. Different studies have taken distinct strategies providing their analysis of treatment response. Some investigations examined the anatomical response to anti-VEGF treatment using OCT, others utilized CNNs employing data from a real-world cohort. It has been observed that the efficacy of anti-VEGF treatment for CNV or cystoid macular edema may be anticipated by analyzing baseline OCT pictures with an area under the curve of 0.81 [36]. In another study, a conditional generative adversarial network (GAN) was employed to create a DL model trained on the retrospective dataset that can generate post-treatment OCT pictures [37]. It is aimed to generate OCT pictures that depict the condition of a patient one month after completing three monthly anti-VEGF loading dosages. A comprehensive model that incorporates baseline OCT, fluorescein angiography, and indocyanine green angiography pictures, instead of relying just on OCT images, demonstrated superior predictive performance for each of PED, SRF, IRF, and subretinal hyperreflective material [38].

**Figure 2:**
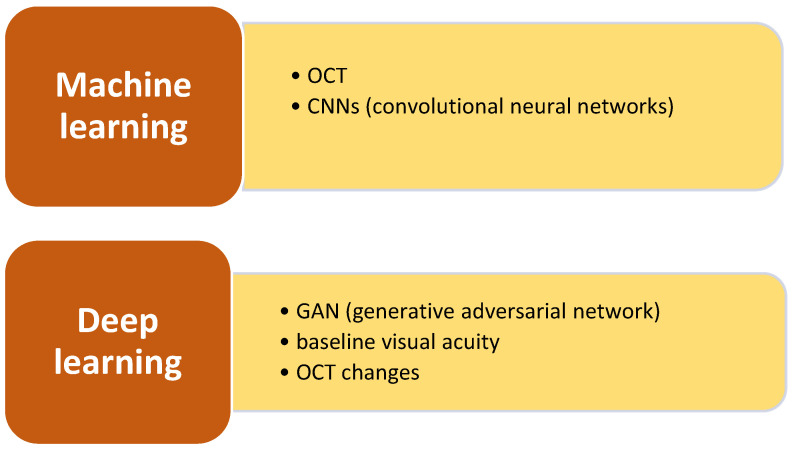
Artificial intelligence models for therapy prediction assessing the anatomical response to antiVEGF.

Some authors employ DL to analyze the Moorfields AMD database in order to investigate the prediction capacity of quantitative OCT parameters on post-treatment visual outcomes [39]. The combination of baseline visual acuity and OCT characteristics accurately predicted the outcomes at 3 months and 12 months after the initial injection, with R2 values of 0.49 and 0.38, respectively. However, when past treatment response (incremental visual acuity and OCT changes) was taken into account, the predictive accuracy significantly improved to R2 values of 0.79 and 0.63, respectively. A group of researchers successfully created a comprehensive DL model to anticipate the treatment needs of patients who are obtaining anti-VEGF on a PRN regimen based on the investigator’s judgment. However, the precise group of patients was not specified [40]. The OCT pictures were examined using established models to measure the amount of fluid. Patients were excluded if there was a disagreement between the model and the investigator on more than three non-injection occurrences over a period of two years [41]. The algorithm classified patients into three categories on the basis of longitudinal images: ‘low’ treatment requirement (up to 5 IVT), ‘mid’ treatment requirement (5 to 15 IVT), and ‘high’ treatment requirement (≥16 IVT). The model showed suboptimal performance in categorizing patients in the second group. However, it attained an area under the curve of 0.85 and 0.81 in binary classifications of low versus all or high vs. all treatment requirements [40]. Nevertheless, this study did not finally establish a direct relationship between these therapy criteria and the resulting eyesight outcomes (Figure 2).

Research has mostly focused on investigating the response to anti-VEGF treatment. This has been performed either indirectly by examining the relationship between OCT parameters and vision outcomes or directly by investigating whether treatment response can be anticipated using retinal pictures [42]. An established concern in the field of ML is that AI models tend to mirror the biases present in the datasets they are trained on. Regrettably, there are rather few extensive datasets, including top-notch spectral-domain OCT data in nAMD, and these are employed by many research groups for the well-known purposes of training, fine-tuning, and testing AI models. Clinical trial populations, characterized by strict inclusion and exclusion criteria, tend to be more homogeneous and less demographically varied compared with real-world populations. On the other hand, patient groups in real-world settings, such as the Moorfields AMD database, exhibit greater diversity in terms of demographics, disease condition, and severity, treatment methods, and the schedule and protocols for OCT imaging [26]. An additional major obstacle to both model building and evaluating a model’s predictive power for treatment needs is the absence of counterfactual data. Each patient features distinct characteristics in terms of the onset of disease, first clinical manifestation, and individualized response to the therapy. Since it is not possible to replicate a specific pre-treatment condition, it is definitely challenging to evaluate the potential outcome of a different treatment strategy for that patient or even forecast their visual outcomes.

The lack of OCT data standards ultimately affects the availability of high-quality datasets for the creation of AI models and the capacity of these models to be used broadly. Consequently, the models developed so far are typically limited to specific devices, which hinders their wider usage in clinical practice where several OCT devices are utilized. To be practical in clinical settings, AI technologies must be specifically developed to be functional and interpretable beyond confined research environments [43].

The goal of obtaining the optimal visual outcome for each individual patient can be achieved through the use of AI-based nAMD therapy predictions in research as well as clinical practice. AI models have the potential to enhance various aspects of clinical trial design, such as patient identification, randomization, selection, and trial analysis adjustments. AI has the potential to enhance standardization and efficiency in picture grading, allowing for analysis on a broader detailed level compared with present technology. AI has the ability to generate ‘synthetic’ treatment arms, which are hypothetical and simulated comparator arms. These arms can be utilized to represent other patient populations or alternative therapies for clinical trials, including sham arms [44].

When it comes to treating nAMD, physicians are restricted in their choices by the best treatment plans that offer the greatest visual improvements while minimizing the burden of treatment. Physicians who adhere to a monthly treatment plan would lack a strong incentive to utilize an artificial intelligence-driven prediction model as an exaggerated illustration. Nevertheless, in the next years, the intricacy of treatment choices is anticipated to rise as the nAMD treatment options expand to potentially encompass novel modes of action, extended-release delivery alternatives, and gene therapy [45].

DL, with thorough OCT image processing, helps doctors determine each patient’s needs as fast and precisely as possible, which will improve patient care in a number of ways. AI can enhance clinicians’ ability to analyze images more effectively, hence increasing the amount of information accessible for therapeutic decision-making, such as identifying tiny features in fundus photographs. For instance, a DL model was created to generate OCT angiography-like images from structural OCT.

## 7. Outcome Prediction Using Artificial Intelligence

Numerous AI techniques have been employed to forecast how patients with nAMD will respond to anti-VEGF treatment. The primary focus of investigations was to forecast the visual outcome after therapy and to anticipate the OCT features following treatment [46]. Rohm et al. utilized ML techniques to make predictions about visual acuity (VA) at 3 and 12 months. The 3-month VA forecast had a mean absolute inaccuracy ranging from 5.5 to 9 letters when compared with the ground truth. Yeh et al. assessed the precision of a new CNN in predicting visual outcomes after 12 months in patients with nAMD. The reported accuracy was 0.936 [47].

Liu et al. utilized GAN to forecast OCT pictures following anti-VEGF therapy for nAMD. According to their research, 92% of the artificial OCT images met the necessary standards for clinical analysis. The predictive accuracy for determining the macular status as either wet or dry was 0.85 [48]. Zhao et al. made an effort to forecast whether patients with nAMD will respond or not respond to short-term anti-VEGF treatment using OCT images [49]. A unique sensitive structure-guided network was utilized, resulting in an 84.6% accuracy in predicting the reaction. The accuracy of this method was comparatively superior to both DL-based systems and experienced ophthalmologists. The work conducted by Lee et al. utilized GAN to make predictions of post-treatment OCT pictures. The inclusion of baseline fluorescein angiography and indocyanine green angiography images resulted in an enhancement of accuracy to a range of 80.7–96.3% [37].

Moon and coworkers specifically examined the variations in fluid levels after therapy among individuals who were administered different anti-VEGF drugs. Two crucial steps are choosing the anti-VEGF molecule and monitoring the fluid status after first loading injections as a significant predictor of long-term effectiveness. In fact, patients showing retinal fluid after the initial loading injection were more likely to have poor long-term visual outcomes and needed more frequent and intensive long-term injections compared with those with no retinal fluid [50]. While both ranibizumab and aflibercept are effective, there are some variations in their efficacy. Aflibercept demonstrated a somewhat higher effectiveness in terms of anatomical improvement, and also a higher rate of clearance of polypoidal lesions, and a bigger decrease in retinal thickness compared with ranibizumab. Shifting from ranibizumab to aflibercept can provide some pros for some people affected by treatment-resistant nAMD, particularly in resolving fluid following the initial loading injections [51]. However, aflibercept has some possible cons. Upon intravitreal injection, a portion of the anti-VEGF drug penetrates the bloodstream, leading to systemic VEGF levels decreasing. Anyway, there is disagreement on whether this distinction (aflibercept vs. ranibizumab) actually results in a medically important effect [52].

Personalized medicine entails tailoring treatment to an individual patient by considering the patient’s characteristics and the nature of the ailment. When evaluating the safety of treating older people with nAMD, ranibizumab may be preferred due to its good systemic risk profile. However, if it is anticipated that ranibizumab treatment would not yield satisfactory results in a patient, aflibercept may also be taken into consideration. When making decisions similar to these, it is crucial to include a highly thorough forecast of the reaction to the treatment. While various biomarkers linked to the response to anti-VEGF medication have been identified, there is currently no established biomarker that can accurately predict the difference in effectiveness between ranibizumab and aflibercept. We are certain that our AI system can assist in predicting the effectiveness of the two medications and so aid in selecting the most suitable anti-VEGF agents for patients (Table 1).

AI has been used to anticipate post-treatment OCT images to detect suitable patients for delivering newly launched medicines to treat nAMD. Ongoing research is being carried out to produce novel drugs for nAMD. Brolucizumab, a novel drug that blocks VEGF-A, was released in 2019. The FDA approved faricimab in 2022. Faricimab is a medication that inhibits VEGF-A and angiopoietin-2. At now, there are ongoing efforts to develop new drugs, such as OPT-30242 and aflibercept 8.0 mg, that can enhance the effectiveness of treating nAMD.

## 8. Future Directions

AI systems can offer enduring cost reductions, particularly in high-volume screening contexts, when cost-effectiveness is taken into account after training and implementation. AI enhances overall resource efficiency by reducing the need for recurrent visits and protracted diagnostic procedures through the automation of the detection process. In addition, AI’s ability to detect nAMD in its initial stages can facilitate the development of timely therapies, thereby reducing the long-term costs associated with the management of advanced conditions. The initial costs of AI systems, which include software, infrastructure, and training, may be substantial; however, these costs are offset by reduced labor costs and improved efficiency as time progresses. Although conventional AMD detection procedures are initially less expensive, they may result in substantial costs over time as a result of the need for frequent follow-up appointments, repetitive examinations, and extended periods for physicians to analyze results. Additionally, traditional methods may result in delayed diagnoses, which could lead to a rise in long-term treatment costs as the disease progresses to more severe stages. The availability of sophisticated diagnostic apparatus may serve as a constraint in resource-constrained environments, thereby rendering AI a more scalable and economically viable option over time.

AI methodologies offer significant advantages over nAMD detection methods in terms of economic viability, efficiency, and precision. AI is an invaluable asset in the management of AMD at the population level and in clinical practice due to its ability to identify early-stage macular changes and automate extensive screening accurately. While AI systems may incur substantial upfront installation costs, their long-term benefits in resource optimization and improved diagnostic precision render them superior to traditional methods.

Cutting-edge technology facilitates the acquisition of a significant amount of imaging data. Consequently, it is expected that AI will progress among the majority of healthcare practitioners across several medical disciplines. The accurate acquisition and evaluation of images are essential for achieving an accurate diagnosis and identifying the most appropriate treatment plan. Advancements in computational resources and machine learning algorithms may improve the clinical practice of physicians and human experts. This may result in a diagnostic technique that is very accurate, reliable, and consistent, exhibiting a high degree of specificity and sensitivity. The expected increase in the availability of medical imaging resources and the reduction in computer technology costs make AI aid essential. Moreover, the extensive variability in the interpretation of imaging results by various examiners, along with the ensuing discrepancies in consensus among retinal specialists, is another factor deserving attention [4]. We are assured that the opportunity to integrate artificial intelligence into routine clinical practice is promising. Deep learning is anticipated to impact clinical practice significantly. Recent research in ophthalmology has demonstrated the validity and accuracy of deep learning algorithms for the early identification, continuous monitoring, and focused therapy of retinal illnesses [57]. Recently developed molecules typically exhibit comparable or even superior results when compared with current agents, but they also might have unforeseen negative occurrences. Therefore, some physicians may adopt a cautious stance towards the utilization of novel medications if the efficacy of existing treatments is deemed satisfactory for treating the ailment. Ranibizumab and aflibercept are currently the two most often utilized FDA-approved drugs for the treatment of nAMD.

Historically, AI has been progressively applied to various areas of ophthalmology, including personalized medicine, to assist in tasks such as selecting appropriate treatments and determining optimal dosage levels. This pattern is anticipated to be adopted in the field of ophthalmology [58]. Some researchers aimed to forecast the variation in post-treatment OCT pictures based on the specific anti-VEGF type, and AI algorithms were applied to forecast alterations in each fluid retinal compartment. Moon conducted a study utilizing horizontal and vertical OCT scan images and employing GAN models, such as cycle GAN and spatial GAN. Additional research will be required to determine the most effective artificial intelligence model for accurately forecasting the treatment outcomes of anatomical conditions, taking into account the individual characteristics of each anti-VEGF medication.

Studies have demonstrated that natural language processing models effectively generate adequate answers to medical questions asked by patients affected by AMD. In a recent study, Johnson et al. demonstrated that the responses generated by Chat-Generative Pre-Trained Transformer (Chat-GPT) were deemed to be very accurate and thorough, with mean scores of “almost completely correct” and “complete and comprehensive” correspondingly [59]. Although still in its early stages, the use of GANs, which are composed of two competing types of deep neural networks, a discriminator and a generator, is showing promising potential applications in ophthalmology, as detailed in an interesting review from You et al. These techniques, including conversion, artifact removal, denoising, and database expansion, can be used in AMD imaging to assist in diagnosis and interpretation [60].

## 9. Conclusions

Models that are based on AI have the potential to improve research and clinical practice in the field of nAMD by allowing for the greatest visual outcomes while minimizing the amount of treatment load for each individual patient. The analysis of OCT, along with other retinal multimodal imaging, having been made possible by the innovative application of AI, has led to a better knowledge of the nAMD and the potential therapeutic responses. Also, there are several modalities by which AI can be utilized in the process of developing, analyzing, and carrying out clinical studies. These applications have the potential to enhance clinical development across the board and boost decision-making confidence, especially in the case of early-stage clinical trials. Additionally, given the complexity of nAMD therapy options, AI may yield formidable tools to guide point-of-care treatment decisions. Anyway, to date, there is still a significant difference between the use of AI for research and for information about clinical practice treatment decisions. The lack of large, reliable datasets reflecting patient variability, pathology, and treatment response is the major obstacle to achieving this goal.

## Figures and Tables

**Table 1:** Machine learning and deep learning models for macular degeneration.

Authors	Model	Dataset
Bogunovic et al. [53]	ML, Random forest classifier	317 eyes from nAMD patients undergoing PRN treatment
Pfau et al. [54]	ML, NGBoost	99 eyes from nAMD patients
Gallardo et al. [55]	ML, Random forest classifier	377 eyes from nAMD patients undergoing T&E treatment
Zheng et al. [56]	DL, U-net DL segmenter, ResNet, iPredict, DenseNet	877 eyes from nAMD patients
Ma et al. [56]	DL, ResNet-34	73 eyes from patients with polypoidal choroidal vasculopathy
Romo Bucheli et al. [40]	DL, DenseNet, RNN trained end to end	350 eyes from nAMD patients undergoing PRN treatment

ML = machine learning; nAMD = neovascular age-related macular degeneration; PRN = pro re nata; T&E = treat and extend; DL = deep learning.

## Data Availability

No new data were created or analyzed in this study. Data sharing is not applicable to this article.

## List of Abbreviations

AI	artificial intelligence
DR	diabetic retinopathy
AMD	age-related macular degeneration
OCT	optical coherence tomography
ML	machine learning
DL	deep learning
ANNs	artificial neural networks
ROP	retinopathy of prematurity
ROC	receiver operating characteristic
AREDS	Age-Related Eye Disease Study
ChatGPT	Chat-Generative Pre-Trained Transformer
IOP	intraocular pressure
FDA	Food and Drug Administration
DM	diabetes mellitus
IDP	Iowa Detection Program
AUC	area under the curve
RMDA	rod-mediated dark adaptation
MAE	mean absolute error
OCTA	OCT angiography
GA	geographic atrophy
MNV	macular neovascularization
RNN	recursive neural network
IVIs	intravitreal injections
RVO	Retinal vein occlusion
BRVO	retinal vein occlusion
CRVO	central retinal vein occlusion
CFPs	color fundus photographs
NPA	nonperfused areas
DNN	deep convolutional neural network
SVM	support vector machines
CDS	clinical decision support
